# How Did Distribution Patterns of Particulate Matter Air Pollution (PM_2.5_ and PM_10_) Change in China during the COVID-19 Outbreak: A Spatiotemporal Investigation at Chinese City-Level

**DOI:** 10.3390/ijerph17176274

**Published:** 2020-08-28

**Authors:** Zhiyu Fan, Qingming Zhan, Chen Yang, Huimin Liu, Meng Zhan

**Affiliations:** 1 School of Urban Design, Wuhan University, 8 Donghu South Road, Wuhan 430072, China; zhiyufan@whu.edu.cn (Z.F.); 2013301540033@whu.edu.cn (M.Z.); 2 Collaborative Innovation Center of Geospatial Technology, 129 Luoyu Road, Wuhan 430079, China; 3 College of Urban and Environmental Sciences, Peking University, Beijing 100871, China; cyangcues@stu.pku.edu.cn; 4 Institute of Space and Earth Information Science, The Chinese University of Hong Kong, Shatin, NT, Hong Kong, China; hmliu@cuhk.edu.hk

**Keywords:** COVID-19, PM pollution, spatiotemporal patterns, spatial correlation analysis, influencing factors, multi-scale geographically weighted regression (MGWR)

## Abstract

Due to the suspension of traffic mobility and industrial activities during the COVID-19, particulate matter (PM) pollution has decreased in China. However, rarely have research studies discussed the spatiotemporal pattern of this change and related influencing factors at city-scale across the nation. In this research, the clustering patterns of the decline rates of PM_2.5_ and PM_10_ during the period from 20 January to 8 April in 2020, compared with the same period of 2019, were investigated using spatial autocorrelation analysis. Four meteorological factors and two socioeconomic factors, i.e., the decline of intra-city mobility intensity (dIMI) representing the effect of traffic mobility and the decline rates of the secondary industrial output values (drSIOV), were adopted in the regression analysis. Then, multi-scale geographically weighted regression (MGWR), a model allowing the particular processing scale for each independent variable, was applied for investigating the relationship between PM pollution reductions and influencing factors. For comparison, ordinary least square (OLS) regression and the classic geographically weighted regression (GWR) were also performed. The research found that there were 16% and 20% reduction of PM_2.5_ and PM_10_ concentration across China and significant PM pollution mitigation in central, east, and south regions of China. As for the regression analysis results, MGWR outperformed the other two models, with R^2^ of 0.711 and 0.732 for PM_2.5_ and PM_10_, respectively. The results of MGWR revealed that the two socioeconomic factors had more significant impacts than meteorological factors. It showed that the reduction of traffic mobility caused more relative declines of PM_2.5_ in east China (e.g., cities in Jiangsu), while it caused more relative declines of PM_10_ in central China (e.g., cities in Henan). The reduction of industrial operation had a strong relationship with the PM_10_ drop in northeast China. The results are crucial for understanding how the decline pattern of PM pollution varied spatially during the COVID-19 outbreak, and it also provides a good reference for air pollution control in the future.

## 1. Introduction

China, as the largest developing country in the world [1], has experienced a rapid process of urbanization and suffered from the accompanying air pollution problems. Among them, particulate matter (PM, mainly including PM_2.5_ and PM_10_) pollution is especially serious in China and has drawn widespread attention from the public [2,3]. Many epidemiological research studies have confirmed that PM pollutants can threat human health, such as increasing the risk of heart disease, lung cancer, and cardiovascular diseases [4,5,6]. It was also reported that the total disability-adjusted life years (DALYs) associated with PM_2.5_ and PM_10_ pollution in China are 7.2 million and 20.66 million, respectively, in 2014–2015 [7]. To improve air quality, the Chinese government has implemented numerous harsh clean air measures, such as shutting down some high polluted factories and eliminating outdated industrial capacities, since 2013 [8,9]. Several research studies found decreasing trends of the annual average PM_2.5_ and PM_10_ concentrations in recent years, especially in some key urban agglomerations, such as the Beijing-Tianjin-Hebei (BTH), the Yangtze River Delta (YRD), and the Pearl River Delta (PRD) [10,11]. Scholars also identified the reduction of industrial emission and the promotion of clean fuels in traffic and residential sectors to be the two dominant factors for the PM pollution reduction in China [12]. However, despite the declining tendencies in some parts of China, the PM pollution is still serious in China given its large concentrations. Specifically, the average concentrations of PM_10_ and PM_2.5_ over China were 89 μg/m^3^ and 43 μg/m^3^ in 2017 [11], which were 4.5 times and 4 times more than the World Health Organization (WHO) guidelines, respectively. [13].

Since the first case reported in Wuhan, a mega city in central China, in December 2019, many more cases of COVID-19 have been found over the country and it has evolved into a global pandemic further. Until 8 April 2020, according to WHO, there had been over 1 million confirmed cases and almost 80 thousand death cases reported worldwide [14]. This new virus is highly contagious and can even spread through aerosol [15]. To cut off the spread of the COVID-19 virus, the Chinese government adopted the strictest self-quarantine measures and listed it as the category B infectious disease on 20 January 2020. People were restricted from traveling outside for entertainment, study, and work and were encouraged to stay at home. In addition, because of the Spring Festival, workers went back to their hometowns, so numerous industrial plants had to be shut down during this period. Under this circumstance, less traffic mobility and industrial operation produced less atmospheric pollutant emissions which were in favor of air quality improvement [16]. Some studies also reported that air pollutants, such as PM_10_, O_3_, and NO_2_, showed significant reductions during the COVID-19 outbreak in some major cities around the world, such as New York, Rome, and Delhi [17,18]. In the Yangtze River Delta Region of China, it was found that the reduction of industrial operations and vehicles’ emissions made a great contribution to air quality improvement during this period [19]. Thus, these control measures are not only necessary for preventing the contagion of COVID-19 virus but also provide an opportunity to examine the effect on PM pollution of traffic restriction and industrial shutdown. In addition, it must be recognized that the pattern of PM pollution reduction tends to vary in regions due to the diversified city response to COVID-19. However, related research studies were mainly focused on local regions or cities, and the national spatiotemporal change pattern of PM was discussed rarely.

In addition to the spatiotemporal variations of PM pollution, the influencing factors driving such variations are also essential for investigating how COVID-19 control measures affected PM pollution. PM pollution related influencing factors can be categorized as natural environmental factors and socioeconomic factors. Some studies have proved that some natural meteorological factors, such as relative humidity, play very important roles in the formation of the substances in PM [20,21]. As for socioeconomic indicators, previous research studies tended to use some indicators from government yearbooks or bulletins, such as secondary industry share, population density, and gross domestic product (GDP) per capita, to reflect the effect of human activities on PM pollution [22,23,24]. Among these indicators, scholars have confirmed that some, like secondary industry share, is significantly correlated with PM pollution, while the influences of other factors, like GDP per capita, are dependent on the development stage of a city or a region [25,26]. Compared with the study periods in previous researches, the period of COVID-19 outbreak is relatively short and it is tough to find appropriate data. For example, traffic mobility reduction, as a vital factor contributing to the PM pollution, is limited by availability and reliability and is hard to measure, especially during a relatively short period. As the development of mobile Internet technology, traffic mobility can be perceived easily by the data from location-based service (LBS) [27]. Moreover, it can present the change of traffic mobility with high temporal resolution, thus possibly being the solution for the short-period research studies, like ours.

In terms of investigation methods, some scholars used classic statistical methods, such as ordinary least square (OLS) model and stochastic impacts by regression on population, affluence, and technology (STIRPAT) model, for relationship analysis [23,28]. These models indicate the overall influence of different factors by some global coefficients and cannot depict the spatial heterogeneity among different geographical objects. To solve this issue, geographically weighted regression (GWR), a local regression method, indicating the influence of factors by many spatial coefficients, were introduced in some research studies [29,30]. However, there are also some disadvantages for GWR. For example, GWR assumes that all influencing factors affect the result at the same scale, which may induce some errors. For the research studies of air pollution underlying mechanism analysis, the relationship between pollutants and different influencing factors usually varies in spatial scales [31]. Therefore, GWR may not perform well for the influencing factors analysis in our research. Recently, an improved model of GWR, multi-scale geographically weighted regression (MGWR), was developed [32]. This model can obtain the optimal bandwidth for each influencing factor and address the issue of imposed scale heterogeneity, which might be the better choice in our research. 

In this study, our goal was to derive the pattern of PM_2.5_ and PM_10_ change by comparing the concentrations in two study periods of 2019 and 2020, respectively, for prefecture cities of China and to further analyze the influencing factors. The study period was set from 20 January to 8 April, when the COVID-19 virus spread seriously in China and related control measures were the strictest during this period in 2020. Firstly, spatial autocorrelation analysis was conducted to recognize the PM concentration change pattern of all prefecture cities in China. Then, for reflecting the traffic mobility reduction, we collected intra-city mobility intensity of Chinese prefecture cities from the Baidu platform and used the average difference between 2019 and 2020 as the indicator for the regression analysis in the next stage. Lastly, MGWR was applied for the relationship investigation between the decline rates of both PM_2.5_ and PM_10_ and the influencing factors, including meteorological factors and socioeconomic factors. 

## 2. Materials and Methods

### 2.1. Data Preparation

#### 2.1.1. Ground-Level PM Measurements Data

**Figure 1:**
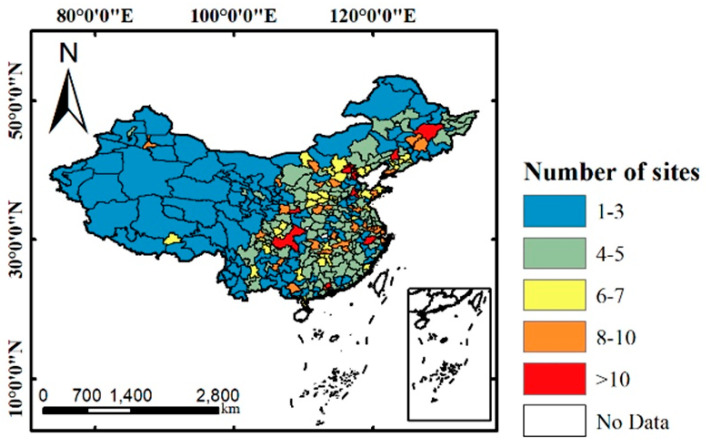
Particulate matter (PM) ground observation sites distribution in China.

Hourly PM_2.5_ and PM_10_ concentration data from 20 January to 8 April in 2018, 2019, and 2020 were downloaded from China National Environmental Monitoring Center (CNEMC, http://www.cnemc.cn). The reason for adding the data of 2018 is to compare the difference in the change between 2019 and 2020 and the change between 2019 and 2018. It includes more than 1500 ground sites established by Chinese government in all 337 prefectural cities of China (excluding Hong Kong, Macau, and Taiwan). The number of sites in each city is given by Figure 1. Among them, there are 232 background sites and the others are non-background sites. At each site, data of PM_2.5_ and PM_10_ were calibrated by automated monitoring systems according to HJ 655-2013 [33]. We first excluded the null data in the original dataset. Then, the daily average PM_2.5_ and PM_10_ concentrations of each site were calculated. Based on daily average data of sites, we further calculated the average concentrations of each city. Due to data deficiency, the average PM_2.5_ and PM_10_ concentrations of 336 cities, in total, for the study period were obtained as final.

Given the distinct concentration variations among different cities of China and two PM pollutants, relative decline rates of the two pollutants, respectively, presenting the temporal changes from 2018 to 2019 and 2019 to 2020, were calculated using formula (1). Here, ${\mathrm{drPM}}_{i}$ is the decline rate of PM pollutants (PM_2.5_ or PM_10_) concentration of city i, and ${\mathrm{PMa}}_{i}$ and ${\mathrm{PMb}}_{i}$ are the average concentrations of the prior year (2018 or 2019) and this year (2019 or 2020) of city i, respectively. Besides, for analyzing the hourly change of PM pollution, average concentrations of each hour were also calculated.

(1) $${\mathrm{drPM}}_{i}=\left({\mathrm{PMa}}_{i}-{\mathrm{PMb}}_{i}\right)/{\mathrm{PMa}}_{i}\;\left(i=1,\;2,\;3\ldots ,\;336\right).$$

#### 2.1.2. Meteorological Data

Multiple meteorological indexes were used for relationship investigation, including relative humidity, air temperature, wind speed, and total precipitation. Data of the meteorological indexes were obtained from the China Meteorological Data Network (CMDN, http://data.cma.cn). This website provides historical daily meteorological observations of all prefectural cities in China. Then, we averaged the daily observations from 20 January to 8 April in both 2019 and 2020 to obtain the mean of each city during the study period. Furthermore, the decline values of four meteorological indicators were derived by using the mean of 2019 minus that of 2020.

#### 2.1.3. Socioeconomic Data

During the outbreak of COVID-19, emission reduction of industry and traffic mobility were the two major reasons reducing air pollution [19]. It has been confirmed that the emission of the industry has a close relationship with the secondary industrial output values (SIOV, i.e., the gross product of secondary industry of each city) [22,34,35]. Therefore, for indicating the decrease of PM concentration caused by the reduction of industrial operation, decline rates of the secondary industrial output values (drSIOV) in the first quarter in 2020 (compared with the first quarter in 2019) of prefectural cities were obtained from the socio-economic operation bulletins. In China, drSIOV were directly announced by each local government statistics office. Limited by data availability, the drSIOV of 264 prefecture cities in total were obtained. Thus, the regression analysis would be conducted on these 264 cities, rather than all 336 cities.

Nevertheless, the traffic mobility reduction can’t be reflected effectively by government documents in this relatively short period of COVID-19 outbreak. Fortunately, mobility data based on LBS provides a solution for this issue. Given the easy contagiousness and the fast spread of COVID-19, some scholars have adopted some mobility data from mobile phones or the Internet to figure out the epidemic and obtained some crucial findings of the spread of COVID-19 in different regions [36,37,38]. Similar to the spread of COVID-19, traffic PM emission also has a close relationship with mobility [39], thus using these mobility data may also work out in detecting the change of PM pollution caused by traffic mobility reduction. We obtained daily intra-city mobility intensity (IMI) data of the corresponding 264 prefectural cities in the study period of 2019 and 2020 from the Baidu platform. Baidu is an Internet company in China and provides the LBS for 195 million people in mainland China. The daily IMI provided by Baidu is the intensity of residents’ traffic travel of a city, which is recognized by origin and destination (OD) pairs of residents based on Baidu LBS data. The higher the index is, the more people go outside (with a relatively long distance) in a city. The mobility data of Baidu also played an important role in other studies about predicting the spread of COVID-19 indicating high data quality [36]. For depicting the change of IMI, we further calculated the decline IMI of each city by using the average IMI of 2019 minus that of 2020.

#### 2.1.4. Variable Summary

To sum up, there are two separate dependent variables and six independent variables. The dependent variables comprise the decline rates of two PM pollutants (drPM_2.5_ and drPM_10_). The independent variables include (1) Meteorological variables, including the decline of relative humidity (dHUM), air temperature (dTEM), wind speed (dWS), and total precipitation (dTP). (2) Socioeconomic variables, including the decline rates of the secondary industrial output values (drSIOV) and the decline of IMI (dIMI). These variables would be applied for the regression analysis investigation further. As mentioned in Section 2.1.3, we only obtained the data of 264 cities in China. Fortunately, these 264 cities are mainly located in the areas where the COVID-19 epidemic was serious during the study period, so the analysis was still representative. Some statistics about the six influencing factors, including dIMI, drSIOV, dTEM, dHUM, dWS, and dTP and two dependent variables, drPM_2.5_ and drPM_10_, in 2019–2020 are shown in Table 1.

### 2.2. Methods

#### 2.2.1. Spatial Autocorrelation

In order to figure out the characteristics of two PM pollutants’ temporal change spatially, spatial autocorrelation analysis was applied. Specifically, the global Moran’s index (Moran’s I) [40], which is widely used for spatial dependence detection, was calculated for drPM_2.5_ and drPM_10_. The calculated formulation is shown as (2) and (3):

(2) $$I_{global}=\frac{\sum_{i=1}^{n}\sum_{j=1}^{n}w_{ij}\left(x_{i}-\bar{x}\right)\left(x_{j}-\bar{x}\right)}{\frac{1}{n}\sum_{i=1}^{n}{\left(x_{i}-\bar{x}\right)}^{2}*\sum_{i=1}^{n}\sum_{j=1}^{n}W_{ij}}\;,$$

(3) $$\bar{x}=\frac{1}{n}\sum_{i=1}^{n}x_{i}.$$

Here, $I_{global}$ is the global Moran’s index, n is the number of all prefecture cities (n = 336). $x_{i}$ and $x_{j}$ are the observations of city i and city j, respectively. $W_{ij}$ represents the neighboring relationship between city i and city j. If city i is adjacent to city j, then $W_{ij}=1$; otherwise, $W_{ij}=0$. The range of global Moran’s index is between −1 and 1. If the value of global Moran’s index is positive (negative), it indicates that there is a positive (negative) correlation among all observations. If the value is closed to 1 (−1), the positive (negative) correlation relationship is strong. In addition, it shows a random distribution of observations in space when the value is closed to 0.

The global Moran’s index can only demonstrate the overall intensity of spatial correlation, so we calculated local Moran’s index [41] further for each city. The formulation is shown in (4):

(4) $$I_{local}^{i}=\frac{x_{i}-\bar{x}}{\frac{1}{n}\sum_{i=1}^{n}{\left(x_{i}-\bar{x}\right)}^{2}}\sum_{j=1,j\neq i}^{n}W_{ij}\left(x_{j}-\bar{x}\right).$$

Here, $\bar{x}$, $x_{i}$, $x_{j}$, $W_{ij}$ are derived the same as mentioned above. $I_{local}^{i}$ is the local Moran’s index of city i.

The significance of $I_{global}$ and $I_{local}^{i}$ can be measured by Z value. The formulation of Z value is given by (5).

(5) $$Z=\;\frac{I-E\left(I\right)}{\surd VAR\left(I\right)},$$

where I is $I_{global}$ or $I_{local}^{i}$, E(I) is the expected value of I, and VAR(I) is the variance of I. For the Z value of global Moran’s index, at the significance level is 0.05, it shows a significant result if the Z value is higher than 1.96 or less than −1.96, while a not significant result otherwise. For the Z value of local Moran’s index, at the significance level is 0.05, if the Z value is higher than 1.96, then it shows that a city with high (low) drPM_2.5_ or drPM_10_ is surrounded by other cities with high (low) drPM_2.5_ or drPM_10_. If the Z value is less than −1.96, then it shows that a city with high (low) drPM_2.5_ or drPM_10_ is surrounded by other cities with low (high) drPM_2.5_ or drPM_10_. If the Z value is between −1.96 and 1.96, it shows that the correlation relationship of a city with surrounding cities is not significant.

#### 2.2.2. Correlation Analysis

Correlation analysis was used for detecting the relationship between the decline rates of PM concentration (drPM_2.5_ or drPM_10_) and influencing factor variables. Here, we selected Pearson Correlation Coefficients as the measurement, and the formulation is given by (6):

(6) $$R_{xy}=\;\frac{\sum_{i=1}^{n}\left(x_{i}-\bar{x}\right)\left(y_{i}-\bar{y}\right)}{\surd \sum_{i=1}^{n}{\left(x_{i}-\bar{x}\right)}^{2}\surd \sum_{i=1}^{n}{\left(y_{i}-\bar{y}\right)}^{2}},$$

where $\bar{x}=\frac{1}{n}\sum_{i=1}^{n}x_{i}$, and $\bar{y}=\frac{1}{n}\sum_{i=1}^{n}y_{i}$. x and y represent the two compared variables. Here, n=264 which is corresponded with the number of cities in regression analysis. $R_{xy}$ is the Pearson Correlation Coefficients and its range is between −1 and 1. It represents a negative relationship between two variables (x and y) when $R_{xy}<0$, and a positive relationship when $R_{xy}>0$. If the absolute value of $R_{xy}$ is close to 1, then it shows a strong correlation relationship between variable x and variable y. If the absolute value of $R_{xy}$ is close to 0, then the correlation relationship is weak.

#### 2.2.3. Regression Analysis and Evaluation

The relationships between the decline rates of PM pollutants and related influencing factors were investigated by OLS, GWR, and MGWR. We would compare the performance of them and use the best model for analyzing further. OLS is a widely used global model for relationship analysis and the formulation can be shown as (7):

(7) $$Y=\beta_{0}+\;\sum_{k=1}^{m}\beta_{k}X_{k}+\varepsilon .$$

where Y is the dependent variable and, specifically, is the PM pollutants decline rate (drPM_2.5_ or drPM_10_). $X_{k}$ is the independent variable k. $\beta_{k}$ is the coefficient of $X_{k}$. ε is the random error item.

GWR is the extension of OLS regression in space. Compared to OLS with a set of constant parameters for all locations, in GWR, each sampling location has their own parameters [42]. Through this, the characteristics of spatial variation can be depicted. The formulation of GWR is given by (8):

(8) $$Y\left(u_{i},v_{i}\right)=\beta_{0}\left(u_{i},v_{i}\right)+\sum_{k=1}^{m}\beta_{k}\left(u_{i},v_{i}\right)X_{k}\left(u_{i},v_{i}\right)+\varepsilon_{i}\left(i=1,\;2,\;3\;\ldots ,\;n,\;n=264\right),$$

where $\left(u_{i},v_{i}\right)$ is the coordinate of the local sampling location. In our research, $\left(u_{i},v_{i}\right)$ of each city is the centroid of all ground PM pollutants observation sites. n is the number of city (n = 264). $\beta_{0}\left(u_{i},v_{i}\right)$ is the intercept, and $\beta_{k}\left(u_{i},v_{i}\right)$ represents the parameter of independent variable $X_{k}$ in location $\left(u_{i},v_{i}\right)$. $\beta_{0}\left(u_{i},v_{i}\right)$ and $\beta_{k}\left(u_{i},v_{i}\right)$ can be estimated by the data from neighboring locations, and the spatial weight of the estimated data can be measured by distance weighting functions. Here, we used Bi-square function in our research for weight calculation, which is given by (9).

(9) $$W\left(i,j\right)=\{\begin{array}{cc} {\left[1-{\left(\frac{d_{ij}}{b_{i}}\right)}^{2}\right]}^{2}, & \;d_{ij}<b_{i}\\ 0, & \;d_{ij}\geq b_{i} \end{array},$$

where $d_{ij}$ is the Euclidean distance between location i and location j. $b_{i}$ is the adaptive bandwidth of location i.

Although GWR can depict the spatial heterogeneity, the assumption that all relationships between the dependent variable and influencing factors operate at the same spatial scale limits the performance and reliability of the regression result. For addressing this issue, MGWR [32], which relaxes the “same spatial scale” assumption and allows specific bandwidths of different independent variables’ relationship with the dependent variable to be optimized, was applied in our research. The formulation of MGWR is given by (10):

(10) $$Y\left(u_{i},v_{i}\right)=\beta_{bw0}\left(u_{i},v_{i}\right)+\;\sum_{k=1}^{m}\beta_{bwk}\left(u_{i},v_{i}\right)X_{k}\left(u_{i},v_{i}\right)+\varepsilon_{i}\left(i=1,\;2,\;3\;\ldots ,\;n,\;n=264\right).$$

Other than $\beta_{bw0}$ and $\beta_{bwk}$, other parts of (10) are the same as (8), where bw0 is the bandwidth of intercept and bwk is the bandwidth of variable k. Therefore, different from GWR, each local parameter $\beta_{k}$ is obtained under the condition of a set of optimal specific bandwidths rather than one bandwidth for all relationships between independent variables and the dependent variable.

We used 3 measurements for evaluating these 3 regression models, including the coefficient of determination (R^2^), the corrected Akaike information criterion (AICc), and the residual sum of squares (RSS). The MGWR model was conducted using the software MGWR2.1 provided by the School of Geographical Sciences and Urban Planning at Arizona State University (https://sgsup.asu.edu).

## 3. Results and Discussions

### 3.1. Overview of PM Pollutants in China

**Figure 2:**
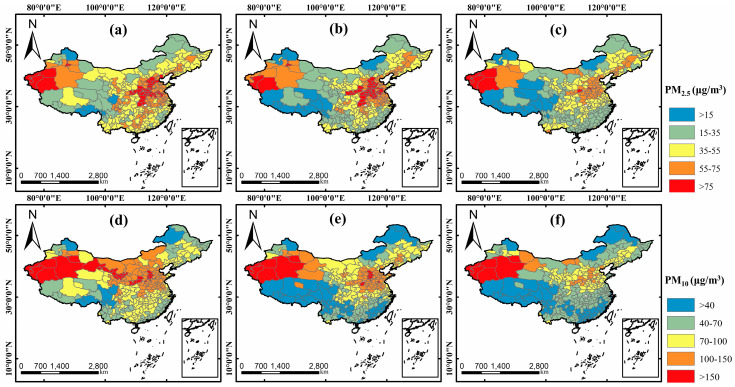
PM concentrations in Chinese prefectural cities of the study period in 2018 ((**a**) for PM_2.5_ and (**d**) for PM_10_); 2019 ((**b**) for PM_2.5_ and (**e**) for PM_10_); and 2020 ((**c**) for PM_2.5_ and (**f**) for PM_10_).

The spatial distribution of PM_2.5_ and PM_10_ in China during the study periods of 2018, 2019, and 2020 are shown in Figure 2. The average concentrations of PM_2.5_ (PM_10_) in study periods from 2018 to 2020 were 52.90 μg/m^3^ (98.47 μg/m^3^), 48.40 μg/m^3^ (82.63 μg/m^3^), and 40.57 μg/m^3^ (66.18 μg/m^3^), respectively, demonstrating a decline trend. Contrast with the decline in the study period of 2019 compared with 2018 (hereafter 2018–2019), the decline in the study period of 2020 compared with 2019 (hereafter 2019–2020) was more notable with decline rates of 16% and 20% for PM_2.5_ and PM_10_, respectively. According to the Chinese Ambient Air Quality Standards (CAAQS) [43], there were only 6 (10), 15 (29), and 20 (73) cities reaching the annual “good” level of PM_2.5_ (PM_10_) standard, respectively (<15 μg/m^3^ for PM_2.5_ and <40 μg/m^3^ for PM_10_) in the study period of 2018, 2019, and 2020. In terms of space, the spatial patterns of both PM_2.5_ and PM_10_ were similar for these 3 study periods with high PM pollution areas in the North China Plain and Xinjiang and low PM pollution areas in coastal and southwest regions. Except some northwest cities located in the desert, it is noted that most cities in China had experienced a huge improvement of PM pollution in 2019–2020. A comparison at national scale between background sites (BS) and non-background sites (NS) was also conducted and is shown in Table 2. Here, Avg_PM_2.5_ and Avg_PM_10_ represent the average PM_2.5_ concentration and PM_10_ concentration, respectively. Results illustrate that there was a consistent decrease in PM concentrations of both two type sites. It is noted that the decreased concentrations of NS in 2019–2020 (about 10 μg/m^3^ for PM_2.5_ and 19 μg/m^3^ for PM_10_, respectively) were much higher than that in 2018–2019 (about 3 μg/m^3^ for PM_2.5_ and 11 μg/m^3^ for PM_10_, respectively), while the decreased concentrations of BS were relatively constant (about 6–9 μg/m^3^ for PM_2.5_ and 18 μg/m^3^ for PM_10_, respectively, for both 2018–2019 and 2019–2020). This is related with the location of background sites. Generally, for better representing the air pollution in large areas, background sites in China were established in the areas that are far away from urban centers. These areas are of relatively low population density and were affected less during the COVID-19 period, so the decreased concentrations in background sites were not significant as that in non-background sites.

**Figure 3:**
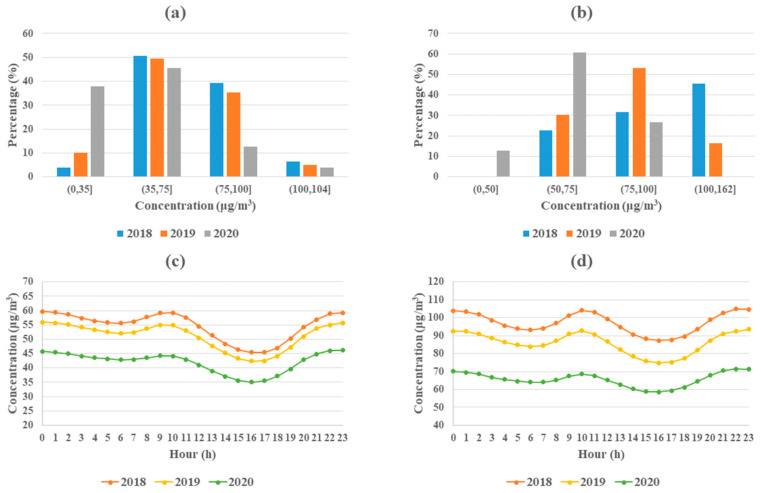
Temporal statistics. (**a**,**b**) The days proportion distribution of daily average PM_2.5_ and PM_10_ concentrations, respectively; (**c**,**d**) The hourly PM_2.5_ and PM_10_ concentrations change, respectively.

In terms of temporal perspectives, the daily average and hourly PM concentration over China was calculated and some related statistics are shown in Figure 3. According to the 24-h average PM_2.5_ standard in CAAQS, there were 4%, 10%, and 38% days in the study periods reaching the “good” level (<35 μg/m^3^) for 2018, 2019, and 2020, respectively. As for PM_10_, there were 12% days in the study period of 2020 reaching the “good” level (<50 μg/m^3^), and there was no day reaching this level in 2018 and 2019. As for the hourly change, both PM_2.5_ and PM_10_ showed a shape of the “W” trend from 0:00 to 23:00 with reaching the peak at 0:00 and 10:00 and reaching the valley at 6:00 and 16:00. In addition, the decreasing concentrations in 2019–2020 were much higher than that of 2018–2019 at all-time points with about 10 μg/m^3^ and 20 μg/m^3^ for PM_2.5_ and PM_10_, respectively.

### 3.2. Spatiotemporal Change Pattern of PM Pollutants in China

#### 3.2.1. Spatiotemporal Variation of PM Pollutants Change

**Figure 4:**
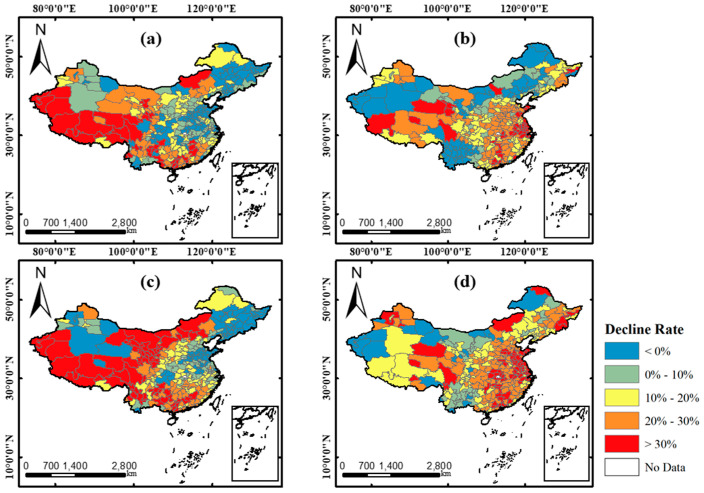
Decline rates of PM_2.5_ and PM_10_. (**a**,**b**) The drPM_2.5_ in 2018–2019 and 2019–2020, respectively. (**c**,**d**) The drPM_10_ in 2018–2019 and 2019–2020, respectively.

The change distribution of PM pollutants, including drPM_2.5_ and drPM_10_, were derived as shown in Figure 4. It can be found that the change distribution of 2019–2020 is totally different from that of 2018–2019 for both PM pollutants. In 2018–2019, the areas with decline rates higher than 30% were mainly located in some western regions, like Tibet and Xinjiang. The PM pollution in north of China especially Heilongjiang, Jilin, Liaoning, Shandong, and Henan were getting worse compared to the study period of 2018. However, the decline rates of 2019–2020 in these northern regions came to more than 20% for both PM pollutants. This is a very interesting phenomenon. These regions always suffer from PM pollution caused by high-emission industrial plants and inappropriate energy consumption structure [44,45], but the COVID-19 control measures became a very effective solution for this issue by accident. In addition, in central and southeast of China, PM pollution also showed a tremendous decline in 2019–2020. As for the comparison of two pollutants, the spatial distribution of drPM_2.5_ and drPM_10_ were similar in 2018–2019 but was observed with some differences in 2019–2020, showing that PM_10_ pollution was improved better than PM_2.5_ pollution in some northern areas. This is because heating from coal burning in north China continued during the COVID-19 outbreak, which still produced much PM_2.5_ [44]. There was a big difference of PM changed patterns between 2018–2019 and 2019–2020 in north western regions, which may be related to some random natural factors, like the sandstorms in spring. As for the city affected most significantly by the COVID-19, Wuhan, drPM_2.5_ and drPM_10_ were 2% and 12% in 2018–2019, respectively, while at 36% and 39% in 2019–2020. This demonstrated a notable PM pollution reduction during the COVID-19 outbreak period.

#### 3.2.2. Spatial Autocorrelation Analysis of PM Pollutants Change

Global Moran’s indexes for drPM_2.5_ and drPM_10_ in 2018–2019 and 2019–2020 were calculated to identify whether the declines of PM_2.5_ and PM_10_ are clustered overall. The result is shown in Table 3. All changes of PM pollutants saw global Moran’s indexes much higher than 0, thus indicating the existence of spatial clustering. The spatial autocorrelation degree of drPM_2.5_ showed a slight increase in 2019–2020 compared to 2018–2019, whereas that of drPM_10_ decreased. Z values of four distribution were higher than 13, which means they all passed the significance test (*p* < 0.001).

**Figure 5:**
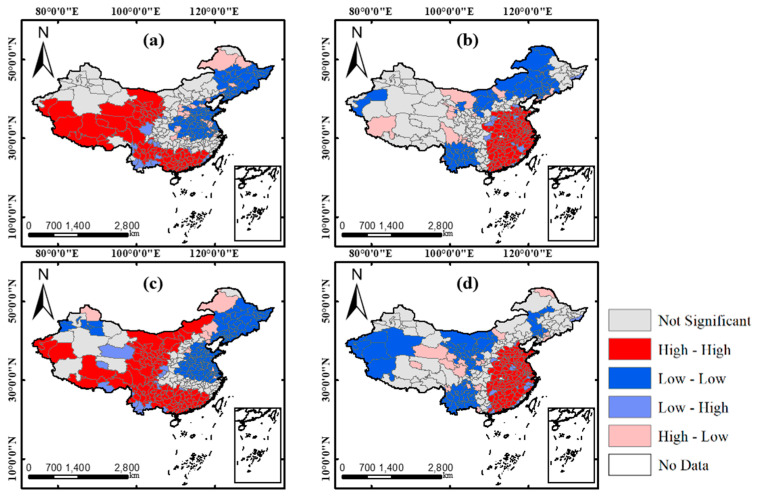
Spatial clusters of drPM_2.5_ and drPM_10_. (**a**,**b**) The results of drPM_2.5_ in 2018–2019 and 2019–2020, respectively; (**c**,**d**) The results of drPM_10_ in 2018–2019 and 2019–2020, respectively.

In order to better display the spatial distribution pattern of drPM_2.5_ and drPM_10_, the clustering results were derived by local Moran’s indexes and were shown in Figure 5. The clustering pattern of local spatial autocorrelation can be categorized as 5 types: “high-high” (high observations surrounded by high observations), “high-low” (high observations surrounded by low observations), “low-high” (low observations surrounded by high observations), “low-low” (low observations surrounded by low observations), and not significant areas. Here, the observation is the drPM, i.e., the decline rate of PM. It can be found that the “high-high” and “low-low” are the main clustering types in all results of the local spatial autocorrelation analysis. Moreover, both two PM pollutants have similar spatial distribution patterns, indicating the close relationship between PM_2.5_ and PM_10_ [46]. The “high-high” clusters were in south, southwest, and some northwest areas, while the “low-low” clusters were in northeast, central, and north areas in 2018–2019. This distribution pattern was also similar with some findings in other researches [47,48,49]. However, in the results of 2019–2020, the “high-high” clusters were mainly located in central, east, and south areas. This was related to the strong and enduring control measures of COVID-19 in these areas. The “low-low” clusters are mainly located in inner-Mongolia, northwest, and southwest areas in 2019–2020. Combined with Figure 4, although it showed a decline trend of PM concentrations in these areas, the degree of decline was not as large as the “high-high” clustering areas and the distribution of the PM pollution decreasing cities was not as dense as that In the “high-high” clustering areas. The “low-low” clustering areas were also the areas affected less by COVID-19 during the study period.

### 3.3. The Relationship between PM Pollutants Change and the Influencing Factors

#### 3.3.1. Overview of Regression Variables

Before regression analysis, correlation analysis based on the Pearson Correlation Coefficient was conducted and the results are shown in Table 4. It can be found that the coefficients between two socioeconomic factors and both decline rates of PM pollutants are positive, which means that the reduction of residents’ travel mobility and industrial production have a strong positive relationship with the drop of PM pollutants. A previous study conducted in North America also confirmed that socioeconomic indicators tend to explain more variations of air pollutants and the findings of ours also are coherent with that view [22]. Additionally, it reflected that dIMI and drSIOV were appropriate as the influencing factors for detecting the changed pattern of PM pollutants caused by COVID-19 control measures. As for the meteorological factors, except dHUM, others have negative relationships with drPM_2.5_ and drPM_10_; so, the higher these meteorological observation values are, the lower PM concentration is in general. This is also consistent with some previous studies [50].

For examining whether the independent variables were fit for linear regressions, multicollinearity was detected using variance inflation factor (VIF) [51]. If the VIF value of an independent variable is higher than 7.5, then there exists multicollinearity, and the variable cannot be used with some other independent variables at the same time. The result is shown in Table 5. It shows that there are no variables with VIF higher than 7.5, so all these variables can be used for linear regression analysis. The abbreviations and the specific meanings can refer to the Section 2.1.4.

#### 3.3.2. Model Performance and Comparison

Three regression models, including OLS, GWR, and MGWR, were conducted based on the normalized independent variables and the performances are summarized in Table 6. The performance of OLS was very poor with R^2^ of 0.227 and 0.217, respectively, for drPM_2.5_ and drPM_10_. Two local regression models performed much better than OLS indicating the superiority of considering spatial heterogeneity. The performance of MGWR further outperforms GWR, with R^2^ of 0.711 and 0.732 for drPM_2.5_ and drPM_10_. In addition to R^2^, the results of RSS and AICc also illustrated the performance of MGWR to be the best among three models. Consequently, the strategy that produces the particular bandwidth for the relationship between each independent variable and the dependent variable in MGWR was effective in our research, and the results of MGWR would be applied for the analysis next.

#### 3.3.3. The Local Relationship between PM Pollutants and the Influencing Factors

MGWR generated many local coefficients of different independent variables and some related statistics are listed in Table 7. It includes three basic statistics including the minimum, maximum, and mean of each variable’s coefficients in the PM_2.5_ model and the PM_10_ model. In addition, the optimal bandwidth (BW) (represents the number of neighboring cities whose data used for constructing local models) and the proportion of coefficients (PC) that passed by significance test (α=0.05) of each independent variable were also shown in Table 7.

The different optimal bandwidths of 6 variables in two MGWR models presented large variations in optimal spatial process scale. The bandwidth of dIMI was the smallest among all variables demonstrating the great spatial non-stationarity of traffic mobility caused by different levels of COVID-19 control measures in different areas. The process scale of drSIOV was larger with the optimal bandwidth of 73 in the PM_2.5_ model and 127 in the PM_10_ model, which exhibited the similar effects of industrial activity reduction spatially. As for meteorological variables, for the PM_10_ model, both the imposed scales of dTEM and dWS were almost as large as the global scale with the number of neighboring of 263 and 229, respectively, while, in the PM_2.5_ model, the optimal scales of all variables were relatively small, except dHUM. This presented that the other three meteorological factors affected the reduction of PM_2.5_ at a relatively local scale compared with that of PM_10_.

In terms of PC, the great difference existed between socioeconomic variables and meteorological variables. The PCs of meteorological variables were all lower than 20% in both PM_2.5_ and PM_10_ models. While for the two socioeconomic variables, it can be found that the estimation of them were significant for more cities especially dIMI with 51% and 56% for the PM_2.5_ model and the PM_10_ model, respectively. Thus, the PM pollutants’ concentration changes in the COVID-19 outbreak period have more closed relationship with the two socioeconomic influencing factors and were slightly affected by meteorology.

**Figure 6:**
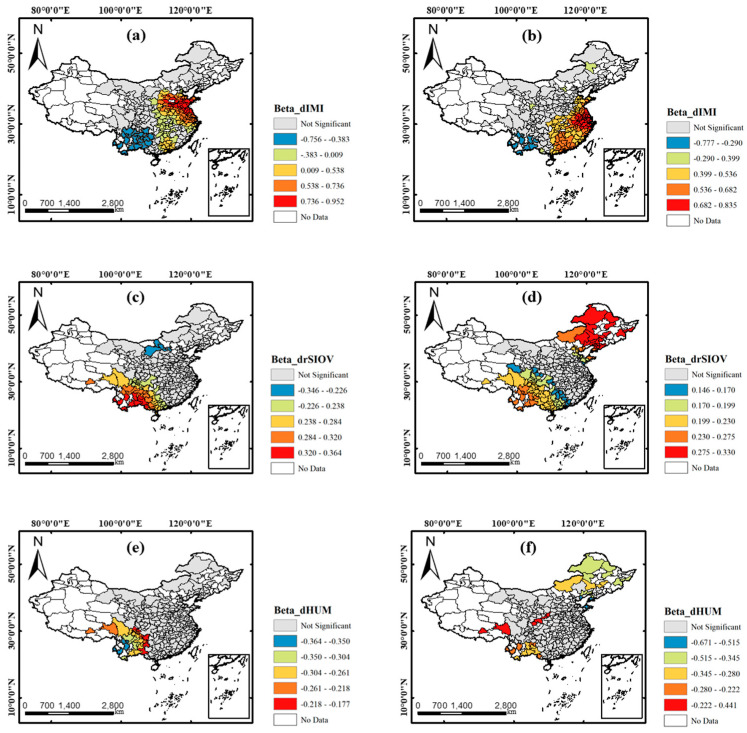
Spatial coefficients of decline of intra-city mobility intensity (dIMI) ((**a**) for the PM_2.5_ model and (**b**) for the PM_10_ model), decline rates of the secondary industrial output values (drSIOV); ((**c**) for the PM_2.5_ model and (**d**) for the PM_10_ model) and dHUM; ((**e**) for the PM_2.5_ model and (**f**) for the PM_10_ model).

Figure 6 shows the spatial coefficients of three variables with the highest PC, including dIMI, drSIOV, and dHUM, in two PM pollutants’ models. The significance of the spatial coefficients was sometimes ignored in some previous researches using local regression, such as GWR and MGWR, and this will lead to questionable results [52]. In our research, despite some “not significant” coefficients, others that passed the *p*-value examination were reliable. In Figure 6, the significant imposed range of different influencing factors varied spatially. The imposed ranges of two socioeconomic factors were much wider than dHUM. Although some meteorological factors like relative humidity are related to the formation of substances in PM, it may affect PM concentration notably at a shorter temporal scale. In addition, it is noted that coefficients of all three factors imposed significantly in southwest regions. On one hand, the cities in these regions are at the relatively primary stage of rapid socioeconomic development and were affected less by the COVID-19 so the influence of socioeconomic factors on PM pollution is remarkable [53]. On the other hand, most cities in these regions with low urbanization rates are surrounded by rainforests so the natural factors, like relative humidity, also play vital roles. The decline of IMI imposed the most significantly impact on the changes of PM pattern with the widest imposed range. In addition, it showed that the coefficient values’ range of dIMI was also the widest in Figure 6. Therefore, the relationship between dIMI and the change of PM during the COVID-19 outbreak is not only the most relevant but also owing the strongest spatial heterogeneity.

From Figure 6a,b, the impacts of traffic mobility reduction on PM pollutants demonstrated great spatial heterogeneity. For both two pollutants, the impacts of traffic mobility were not significant in northwest and northeast regions. In addition, it saw a negative relationship with PM concentration in southwest regions, such as Yunnan and Guizhou. There are two aspect reasons for explaining this. On one hand, the PM pollutants reduction during the COVID-19 outbreak period was not obvious and even showed increased trends in these regions, which may be related with some exterior pollutants from southeast Asian countries [54]. On the other hand, the population density in these regions is relatively low, which means the reduction of residents’ travel did not contribute too much to the PM pollutant emission [29]. Thus, despite a little PM decrease caused by traffic mobility reduction, the overall PM pollution did not decrease too much. On the contrary, traffic reduction had a notable effect on PM pollution drop in central, south, and east of China. It can be found that a decrease of 0.1 in IMI would lead to a decline in PM concentrations by higher than 5% in these regions. These regions with the highest population density suffered the COVID-19 control measures for a long time and both the decreases of IMI and PM pollution were very significant. Although the decline of traffic mobility made a great contribution to PM decline during the COVID-19 outbreak, it must be recognized that it is not a long-term effective solution. The government should further widely promote the application of electrical and hydrogen vehicles and encourage residents to use more public transportation [55,56]. There were also some differences between the two PM pollutants. Except for the south regions, the cities with high coefficients in the PM_2.5_ model were mainly distributed in eastern regions, such as Jiangsu and Zhejiang, while those in the PM_10_ model were mainly in central regions, such as Henan and Shandong. This may be related to the different main emissions of traffic [57,58]. Compared with using complex traditional traffic emission inventories, it is more convenient to apply intra-city mobility intensity obtained based on real-time LBS data to measure the influence of traffic mobility on PM pollution during a relatively short time like the COVID-19 outbreak period. In addition, there is also some uncertainty about the applicability of traditional emission inventories [59]. Thus, in terms of presenting the relationship of traffic mobility and PM pollution, LBS data, like IMI, may be the best choice in our research.

Figure 6c,d present the spatial local coefficients of drSIOV. The reduction of industrial production led to a great impact on PM_10_ drop in northeast China where is the basis of the Chinese heavy industry with very large secondary industry share. The local economic development mainly relies on mining, metal process, and machine manufacturing, which are the main sources of PM_10_ pollution [60]. The COVID-19 control measures hindered the industrial operation thus making a significant contribution to PM_10_ pollution improvement. In addition, the industrial operation has a strong relationship with PM_2.5_ pollution concentration in southwest regions where the industrial operation was less affected and PM_2.5_ pollution also showed a slightly increasing trend. In recent years, due to some restricted policies of central government on air pollution, many industrial enterprises were transferred from some developed regions like east of China to the southwest and this led to some serious air pollution issues [53]. Despite relative low PM concentrations in the COVID-19 outbreak, local government in southwest of China should be alert to the deterioration of PM pollution caused by industrial emissions. For both two pollutants’ models, the “not significant” cities are mainly in northwest, south, and east regions. Due to the advanced technology and economy, industrial operation in south and east regions may not be closely related to PM pollutants. This is also corresponded with the EKC model (as the economic development, the pollution became worse first and then is alleviated) and some other previous findings in certain areas and other countries, like Canada [53,61,62]. As for northwest regions, the reason may be related to some other natural factors, such as the deserts in inner-Mongolia [63].

Figure 6e,f show the local coefficients of dHUM. It can be found that there was mainly a negative relationship with PM pollution for relative humidity in the cities located in the forest and plateau. In addition, the relationship between relative humid with PM_10_ is positive for cities located in the Qinling Mountains and west of Sichuan. For dHUM, there are only 12% and 14% of PC for PM_2.5_ and PM_10_, respectively, although it is the factor with the highest PC among all four meteorological factors.

## 4. Conclusions

In this study, we obtained the changed pattern of PM_2.5_ and PM_10_ concentration at city-scale in China during the COVID-19 outbreak compared with the same period of 2019 by spatial autocorrelation analysis. In addition, an improved model of GWR, MGWR, was applied for detecting the influence of four meteorological factors and two socioeconomic factors on the drop of PM pollution. The main findings are as follows:

(1) The decline changes of both PM_2.5_ and PM_10_ presented a totally different spatiotemporal pattern in 2019–2020 compared with that of 2018–2019. In the result of 2018–2019, obvious “low-low” clustering patterns were found in north, central and east regions of China, whereas, in 2019–2020, it showed strong clustering patterns of high decline rates of PM_2.5_ and PM_10_ in these regions

(2) IMI derived from Baidu and secondary industrial output values derived from local government bulletins were adopted to measure the influence of the decline of traffic mobility and industrial operation. These two variables showed an obvious positive relationship with the decline of PM_2.5_ and PM_10_.

(3) MGWR outperformed OLS and GWR, with R^2^ of 0.711 and 0.732 for drPM_2.5_ and drPM_10_, respectively. This reflected that the strategy that releases the restriction of constant scales was very effective in our research. Thus, the results of MGWR were applied in the analysis of influencing factors.

(4) In the results of MGWR, compared with meteorological variables, the decline of IMI and the decline rate of secondary industrial output values had a more significant impact on PM pollution reduction. It showed that the reduction of traffic mobility caused more reduction of PM_2.5_ in east China, such as Jiangsu and Zhejiang, while more reduction of PM_10_ in central regions, such as Shandong and Henan, were observed. In addition, it was found that the reduction of secondary industrial output values caused more PM_2.5_ drop in southwest regions of China and more PM_10_ drop in northeast of China.

This study also has some limitations. First, limited by data, this study only adopted the data of 264 prefectural cities, which may cause some biases. Second, there are some other influencing factors, such as biomass burning, sand dust, and biogenic and biological PM, were not involved. Third, most PM observation sites are in urban areas while sparsely distributed in rural areas. This may also induce some uncertainty to the results. Last, some other meteorological factors, like atmosphere stability, which are also related with PM are not involved in this study, and we will consider these when data is available.

In general, although the COVID-19 control measures restricted travel freedom and economic development and forced people to conduct tele-work and tele-study, it provided an excellent and precious opportunity for observing PM pollution improvement. The findings of our research reflect the spatiotemporal PM pollution decline pattern and the impact of related influencing factors, thus being helpful for air pollution mitigation and control for the government. However, it also should be recognized that, despite the remarkable reduction, the concentrations of PM_2.5_ and PM_10_ were several times higher than the WHO standards during the COVID-19 outbreak. Therefore, there is still a long way to go to limit the PM pollution of China in the future.

## Figures and Tables

**Table 1:** Some information about variables.

Variable.	Min	Max	Mean	Std
drPM_2.5_ (%)	−58.803	42.025	16.213	15.495
drPM_10_ (%)	−19.822	45.424	21.739	10.842
dTEM (℃)	−2.06	2.84	−0.572	0.905
dHUM (%)	−19.051	11.926	−2.35	6.312
dWS (m/s)	−0.8	0.598	−0.004	0.173
dTP (mm)	−3.538	1.98	−0.342	0.738
drSIOV (%)	−0.142	0.537	0.089	0.108
dIMI (-)	−1.29	2.95	0.971	0.507

**Table 2:** Average PM concentration comparison between background sites (BS) and non-background sites (NS).

Sites	BS in 2018	BS in 2019	BS in 2020	NS in 2018	NS in 2019	NS in 2020
Avg_PM_2.5_ (μg/m^3^)	50	44	35	55	52	42
Avg_PM_10_ (μg/m^3^)	86	68	51	96	85	66

**Table 3:** Global Moran’s indexes for the decline rates in 2018–2019 and 2019–2020 of PM pollutants.

Change	Global Moran’s Index	Z Value
drPM_2.5_ for 2018–2019	0.453	13.812
drPM_10_ for 2018–2019	0.628	19.070
drPM_2.5_ for 2019–2020	0.492	15.028
drPM_10_ for 2019–2020	0.452	13.806

**Table 4:** Pearson Correlation Coefficients between 6 independent variables and 2 dependent variables.

Variable.	dTEM	dHUM	dWS	dTP	dIMI	drSIOV
drPM_2.5_	−0.317 ***	0.177 **	−0.236 ***	−0.205 ***	0.258 ***	0.214 **
drPM_10_	−0.220 ***	0.015	−0.134 *	−0.254 ***	0.376 ***	0.245 ***

Note: *** means the result satisfies the significance level test of α=0.001, ** means the result satisfies the significance level test of α=0.01 and * means the result satisfies the significance level test of α=0.05.

**Table 5:** Variance inflation factors (VIFs) among independent variables.

Variable.	VIF
dHUM	1.24
dTEM	1.21
dWS	1.03
dTP	1.09
drSIOV	1.20
dIMI	1.48

**Table 6:** Model performance of ordinary least square (OLS), geographically weighted regression (GWR), and multi-scale geographically weighted regression (MGWR).

Model.	R^2^		RSS		AICc	
Dependent Variable	drPM_2.5_	drPM_10_	drPM_2.5_	drPM_10_	drPM_2.5_	drPM_10_
OLS	0.227	0.217	204.167	206.591	697.613	701.030
GWR	0.658	0.653	90.367	91.611	600.388	602.003
MGWR	0.711	0.732	76.312	70.747	569.028	545.205

**Table 7:** Some information about the coefficients in two MGWR models. BW is the optimal bandwidth of each variable, and PC is the proportion of coefficients passed by significance test (α=0.05).

Variable.	PM_2.5_ MGWR Model					PM_10_ MGWR Model				
	Min	Max	Mean	BW	PC	Min	Max	Mean	BW	PC
dTEM	−0.07	0.19	0.01	127	2%	−0.01	0.02	0.004	263	0%
dHUM	−0.36	0.10	−0.04	94	12%	−0.67	0.44	−0.04	49	14%
dWS	−0.62	0.49	−0.02	45	14%	−0.03	0.17	0.1	229	0%
dTP	−0.67	0.18	−0.10	43	8%	−0.48	0.28	−0.02	45	3%
drSIOV	−0.35	0.36	0.09	73	23%	0.09	0.33	0.16	127	45%
dIMI	−0.76	0.95	0.32	43	51%	−0.78	0.84	0.23	43	56%
